# Esophageal surgical Apgar score (eSAS): A predictor for postoperative morbidity in patients undergoing neoadjuvant therapy and esophagectomy

**DOI:** 10.1111/1759-7714.15246

**Published:** 2024-02-23

**Authors:** Qin Wang, Chi Zhang, Chen Qi, Yong Qiang, Zheng Zhang, Fei Xu, Yi Shen

**Affiliations:** ^1^ Department of Cardiothoracic Surgery, Jinling Hospital Medical School of Nanjing University Nanjing China; ^2^ Department of Cardiothoracic Surgery, Jinling Hospital, School of Medicine Southeast University Nanjing China; ^3^ Department of Cardiothoracic Surgery, Jinling Hospital, School of Clinical Medicine Nanjing Medical University Nanjing China

**Keywords:** esophageal cancer, esophagectomy, esophagectomy surgical Apgar score, morbidity

## Abstract

**Background:**

The surgical Apgar score (SAS) quantifying three intraoperative indexes has been confirmed to be significantly associated with postoperative morbidity and prognosis in many surgical specialties. However, there are great limitations in its application for esophageal cancer (EC). This study aimed to assess the predictive capability of esophagectomy SAS (eSAS) in determining postoperative morbidity and overall survival (OS) in EC patients who had undergone neoadjuvant therapy.

**Methods:**

A retrospective evaluation was conducted on a cohort of 221 patients in which surgery‐ and tumor‐related data were extracted and analyzed. Major morbidity was defined as complications meeting the criteria of Clavien‐Dindo classification III or higher during hospitalization. Univariate and multivariate analyses were performed to identify potential risk factors for major morbidity. Kaplan–Meier analysis was utilized to calculate the OS and relapse‐free survival (RFS).

**Results:**

The results exhibited that eSAS demonstrated potential predictive value for postoperative morbidity with an optimal cutoff value of 6. The eSAS and diabetes mellitus were two independent risk factors for the major morbidity; however, no correlation between the eSAS and the OS or RFS was detected.

**Conclusion:**

The eSAS could be used as a predictor of major morbidity, while it was not correlated with OS and RFS.

## Graphical abstract

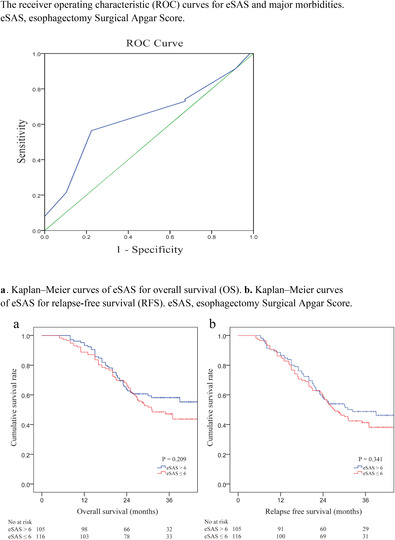

## INTRODUCTION

Esophageal cancer (EC) ranks the sixth most prevalent cause of cancer‐related mortality worldwide and stands as the second most fatal gastrointestinal malignancy.^1^ The majority of EC cases are diagnosed at an advanced stage, with a five‐year survival rate scarcely surpassing 40%.^2^, ^3^ Surgical intervention remains the primary course of treatment, and numerous studies have indicated that postoperative resection subsequent to neoadjuvant therapy can ameliorate the prognosis of patients with early‐stage localized and metastatic EC.^4^, ^5^, ^6^ In 2019, the National Comprehensive Cancer Network (NCCN) guidelines recommended that locally advanced EC patients could derive immense benefits from neoadjuvant therapy.^7^ Despite substantial advancements in modern surgical techniques, the postoperative morbidity rate for esophagectomy still hovers around 60% due to surgical stress, as evidenced by previous studies.^8^, ^9^ Given that surgical resection remains the most pivotal treatment modality for EC, the occurrence of postoperative complications emerges as a significant adverse factor impacting patients' overall survival (OS). In light of the foregoing, the accurate prediction and proactive management of these complications play a pivotal role in optimizing the treatment outcomes for EC patients.^10^, ^11^

There have been various studies which have focused on preoperative parameters such as the prognostic nutritional index (PNI), neutrophil‐to‐lymphocyte ratio (NLR), platelet‐to‐lymphocyte ratio (PLR) and controlling nutritional status (CONUT) to improve clinical outcomes via better patient selection, and to enhance risk‐stratified comparative analyses for quality assessments.^12^, ^13^, ^14^, ^15^ However, there have been studies on scoring systems that stratified surgical risk factors which contribute to adverse peri‐ and postoperative outcomes.^16^, ^17^ Importantly, their shortages derived from calculation boundedness as an analytical model. To conclude, it is imperative to explore a new prognostic index based on intraoperative parameters that can accurately predict postoperative modalities and prognosis in EC patients.

A comprehensive 10‐point scoring system known as the surgical Apgar score (SAS) was introduced by Gawande et al. in 2007 based on three intraoperative parameters: estimated blood loss (EBL), lowest heart rate (L‐HR), and lowest mean arterial blood pressure (L‐MAP).^18^ The SAS had been widely recognized for its significant association with postoperative morbidity and prognosis across various surgical specialties.^19^, ^20^, ^21^ However, the value of the SAS in EC remains obscure and disputable. The aim of the present study was to elucidate the mathematical relationship between the esophageal surgical Apgar score (eSAS) and postoperative morbidity or prognosis.^22^, ^23^

## METHODS

### Patient characteristics

A total of 238 patients (≥18 years) with esophageal cancer who had undergone neoadjuvant therapy and esophagectomy at the Department of Oncology and Cardiothoracic Surgery of Jinling Hospital were included in the study, and 17 were excluded with the condition of two‐stage esophagectomy or transhiatal esophagectomy. This study was conducted in accordance with the Helsinki Declaration of 1964 and the latest version and met the ethical standards of the responsible committee on human experimentation of Jinling Hospital. Detailed interpretation of the study protocol was explained to participants with informed consent being obtained from all of them.

### Data collection

Patient data including age, gender, smoking history and comorbid disease (cardiovascular disease, pulmonary disease, chronic liver disease, chronic renal disease and diabetes mellitus) were extracted and analyzed. The tumor‐related data including tumor histology, tumor location and post‐neoadjuvant (ypTNM) stage was according to the eighth American Joint Commission on Cancer (AJCC) Cancer Staging Manual.^24^ The surgery‐related data included the American Society of Anesthesiologists (ASA) score, the type of surgery, the duration of operation, eSAS and blood transfusion. L‐MAP and L‐HR were collected and assigned points according to the method proposed by Regenbogen et al.^25^ The ranges used to assign points for EBL in the original SAS scoring system were adjusted, with the cutoff points based on quartile values of EBL in our patient cohort. The median 200 mL EBL (range, 100–1100 mL; interquartile range, 150–275 mL) was consistent with previous studies^26^ (Table 1).

**TABLE 1:** Esophagectomy surgical Apgar score (eSAS).

Variables	0 point	1 point	2 points	3 points	4 points
EBL (mL)	>275	201–275	151–200	≤150	–
L‐MAP (mmHg)	<40	40–54	55–69	≥70	–
L‐HR (beats/min)	>85	76–85	66–75	56–65	≤55

Abbreviations: EBL, estimated blood loss; eSAS, esophagectomy surgical Apgar score; L‐HR, lowest heart rate; L‐MAP, lowest mean arterial blood pressure.

### Definition of outcomes

Complications meeting the definition for Clavien‐Dindo classification III or higher which occurred during hospitalization were categorized as major morbidity, including severe anastomotic, respiratory, cardiac, recurrent nerve palsy, chylothorax complications.^27^, ^28^ Mild complications of anastomotic, respiratory and some other types were excluded. The OS was measured from the day of diagnosis to the day of death or last follow‐up, while relapse‐free survival (RFS) was measured from the day of diagnosis to the date of recurrence or last follow‐up.

### Treatment strategy

The regimen of neoadjuvant therapy was selected using the guidelines of NCCN for esophageal and esophagogastric junction cancers.^7^ Neoadjuvant chemotherapy included two courses of paclitaxel plus cisplatin (TP) or 5‐fluorouracil plus cisplatin (FP). Neoadjuvant chemoradiotherapy included two cycles of chemotherapy with sequential or concurrent radiotherapy. The radiotherapy dose was 41.4–50.4 Gy. Among the 19 patients with upper esophageal cancer, 17 underwent neoadjuvant chemoradiotherapy. The interval between neoadjuvant therapy and surgery was no less than 3 weeks. All patients underwent normalized adjuvant therapy if necessary. McKeown esophagectomy or Ivor Lewis esophagectomy combined with two‐or‐three field lymph node dissection was performed in a standardized manner.

### Statistical analysis

The optimal cutoff value and discrimination ability of eSAS for postoperative morbidity was evaluated according to receiver operating characteristic (ROC) curve analysis. Categorical variables were analyzed using Pearson's chi‐square test or Fisher's exact test, and continuous variables were analyzed using the student's *t*‐test. Risk factors for postoperative morbidity were assessed using univariate logistic regression analysis, and factors with a *p*‐value less than 0.05 were performed in multivariate analysis to calculate adjusted odds ratios (ORs) and 95% confidence intervals (CIs). Kaplan–Meier was used to calculate the OS and RFS curves. Statistical analyses were performed using SPSS 27.0. *p* < 0.05 was considered statistically significant.

## RESULTS

A total of 211 patients who underwent neoadjuvant therapy and esophagectomy at our institution were retrospectively evaluated. The incidence of major morbidity was 26.2%, median age was 67 ± 7.56 years, and 176 patients (79.6%) were male.

### Cutoff value of eSAS

**FIGURE 1:**
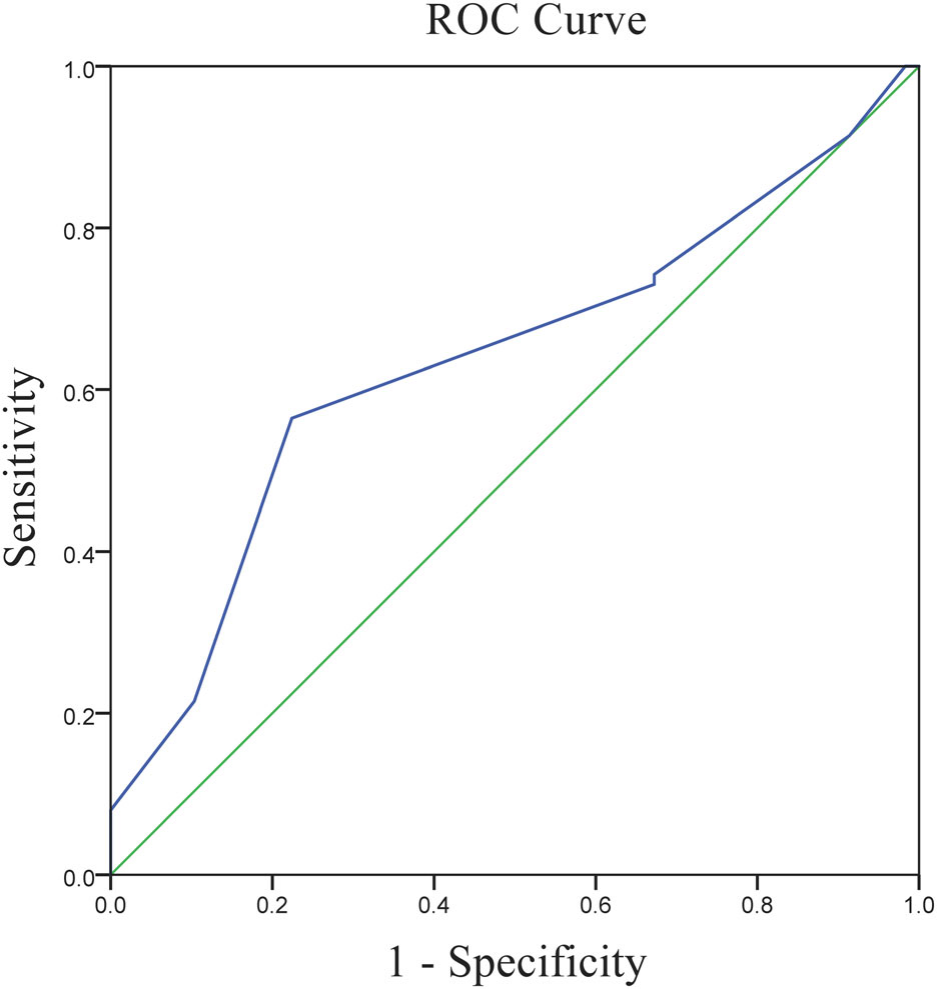
The receiver operating characteristic (ROC) curves for eSAS and major morbidities. eSAS, esophagectomy surgical Apgar score.

**FIGURE 2:**
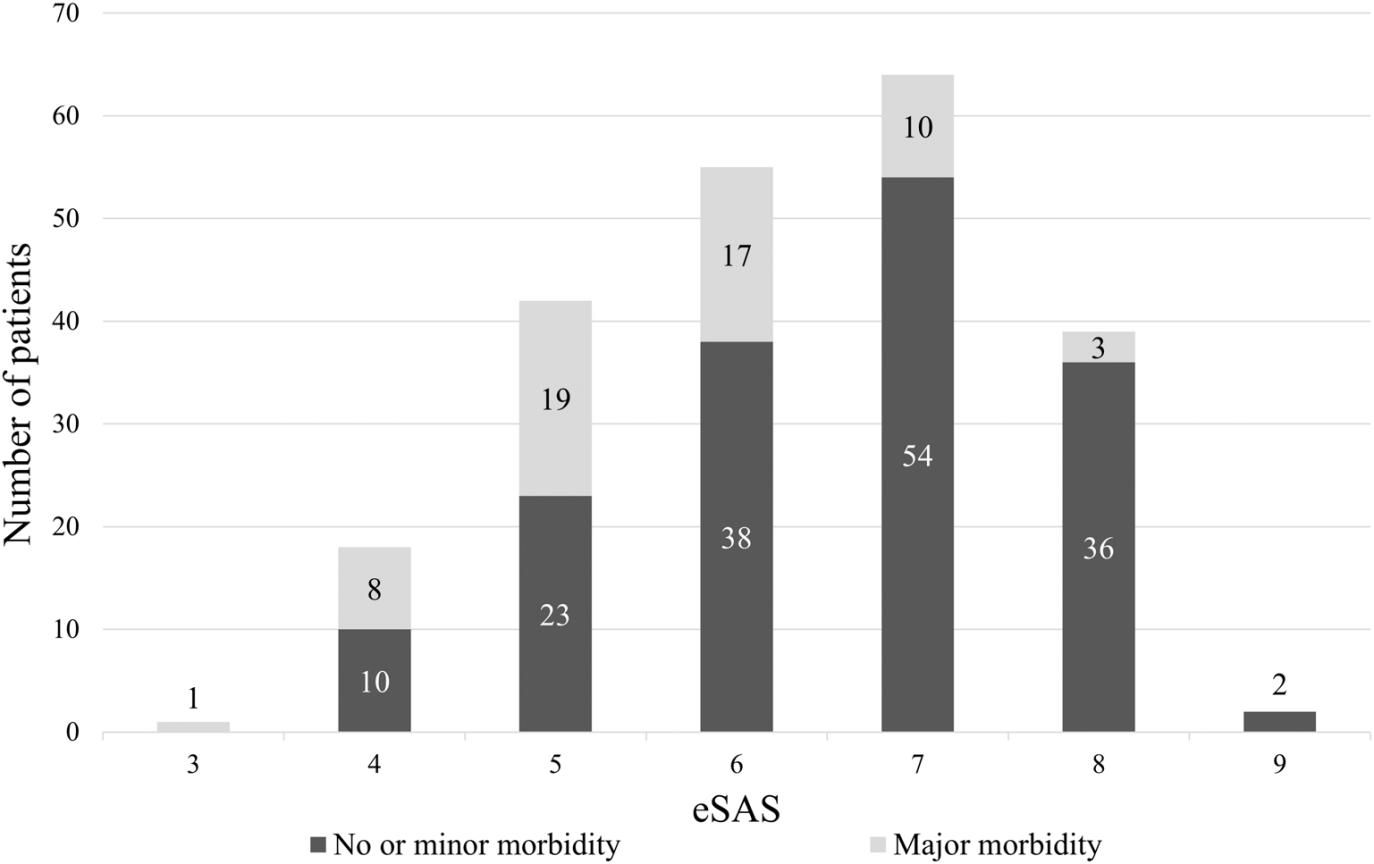
Distribution of patients by the eSAS. eSAS, esophagectomy surgical Apgar score.

According to the ROC curve (Figure 1), eSAS had an obvious discrepancy in postoperative morbidity prediction (*p* = 0.002) and the optimal cutoff value was six. In the score of six or less group (*n* = 116), 38.8% of patients underwent major morbidity while the percentage was only 12.4% in the score above six group. The distribution of patients by eSAS is shown in Figure 2.

### Univariate and multivariate analyses of postoperative morbidities

Univariate analysis showed the occurrence of major morbidity was significantly correlated with age of 65 years or older (*p* = 0.047), ASA classification III–IV (*p* = 0.037), diabetes mellitus (*p* = 0.022), open esophagectomy (*p* = 0.026), EBL (*p* = 0.024) and eSAS (*p* < 0.001). To the contrary, no association was found between major morbidity and gender, smoking, cardiovascular disease, pulmonary disease, chronic liver disease, chronic renal disease, histology, tumor location, ypTNM stage, operation time, and blood transfusion or not. Estimated blood loss was excluded from the multivariate model due to its statistical correlation with eSAS (Table 2). Multivariate analysis demonstrated that eSAS (OR = 3.794, *p* < 0.001) and diabetes mellitus (OR = 2.812, *p* = 0.026; Table 3) were two independent risk factors for major morbidity, with mild heterogeneity. For detailed complications, a low eSAS was associated with anastomotic leakage (AL) (*p* = 0.006), surgical site infection (*p* = 0.017), and reintubation (*p* = 0.027). No significant association was shown in eSAS and other complications (Table 4).

**TABLE 2:** Clinicopathological, operative and biochemical variables associated with anastomotic leakage.

Variables	*N* (%)	Number of patients (% of a subgroup)		*p*‐value
		Major morbidity	No or minor morbidity	
Total	221	58 (26.2)	163 (73.8)	
Age (years)				
<65	89 (40.3)	17 (29.3)	72 (44.2)	0.047
≥65	132 (59.7)	41 (70.7)	91 (55.8)	
Gender				
Male	176 (79.6)	50 (86.2)	126 (77.3)	0.15
Female	45 (20.4)	8 (13.8)	37 (22.7)	
ASA score				
I/II	207 (93.7)	51 (87.9)	156 (95.7)	0.037
III/IV	14 (6.3)	7 (12.1)	7 (4.3)	
Smoking				
Never	107 (48.4)	24 (41.4)	83 (50.9)	0.21
Pre‐/current	114 (51.6)	34 (58.6)	80 (49.1)	
Comorbid disease				
Cardiovascular disease				
None	213 (96.4)	54 (93.1)	159 (97.5)	0.12
History	8 (3.6)	4 (6.9)	4 (2.5)	
Pulmonary disease				
None	187 (84.6)	50 (86.2)	137 (84.0)	0.70
History	34 (15.4)	8 (13.8)	26 (16.0)	
Chronic liver disease				
None	200 (90.5)	52 (89.7)	148 (90.8)	0.79
History	21 (9.5)	6 (10.3)	15 (9.2)	
Chronic renal disease				
None	192 (86.9)	47 (81.0)	145 (89.0)	0.13
History	29 (13.1)	11 (19.0)	18 (11.0)	
Diabetes mellitus				
None	194 (87.8)	46 (79.3)	148 (90.8)	0.022
History	27 (12.2)	12 (20.7)	15 (9.2)	
Histology				
SCC	196 (88.7)	54 (93.1)	142 (87.2)	0.40
AC	16 (7.2)	2 (3.4)	14 (8.6)	
ASC	3 (1.4)	0 (0)	3 (1.8)	
Other	6 (2.7)	2 (3.4)	4 (2.4)	
Tumor location				
Upper	19 (8.6)	6 (10.4)	13 (8.0)	0.35
Middle	120 (54.3)	35 (60.3)	85 (52.1)	
Lower	82 (37.1)	17 (29.3)	65 (39.9)	
ypTNM stage				
Stage I	102 (46.1)	25 (43.1)	77 (47.2)	0.63
Stage II	29 (13.1)	9 (15.5)	20 (12.3)	
Stage III	83 (37.6)	21 (36.2)	62 (38.1)	
Stage IVA	7 (3.2)	3 (5.2)	4 (2.4)	
Pathological T stage				
ypT0	81 (36.7)	18 (31.0)	63 (38.7)	0.51
ypT1	21 (9.5)	4 (6.9)	17 (10.4)	
ypT2	31 (14.0)	11 (19.0)	20 (12.3)	
ypT3	86 (38.9)	24 (41.4)	62 (38.1)	
ypT4	2 (0.9)	1 (1.7)	1 (0.6)	
Pathological N stage				
ypN0	132 (59.7)	34 (58.6)	98 (60.1)	0.54
ypN1	59 (26.7)	17 (29.3)	42 (25.8)	
ypN2	23 (10.4)	4 (6.9)	19 (11.7)	
ypN3	7 (3.2)	3 (5.2)	4 (2.4)	
Operation type				
MIE	175 (79.2)	40 (69.0)	135 (82.8)	0.026
OE	46 (20.8)	18 (31.0)	28 (17.2)	
Duration of operation (minutes)		221.14 ± 58.08	229.38 ± 65.63	0.40
EBL (mL)		246.90 ± 150.929	210.55 ± 82.307	0.024
Blood transfusion				
NO	212 (95.9)	54 (93.1)	158 (96.9)	0.21
YES	9 (4.1)	4 (6.9)	5 (3.1)	
eSAS				
>6	105 (47.5)	13 (22.4)	92 (56.4)	<0.001
≤6	116 (52.5)	45 (77.6)	71 (43.6)	

*Note*: Data are presented as *n* (%) or mean ± standard deviation (SD).

Abbreviations: AC, adenocarcinoma; ASA score, American Society of Anesthesiologists score; ASC, adenosquamous carcinoma; EBL, estimated blood loss; eSAS, esophagectomy surgical Apgar score; MIE, minimally invasive esophagectomy; OE, open esophagectomy; SCC, squamous cell carcinoma; ypTNM, post‐neoadjuvant pathological TNM.

**TABLE 3:** Univariate and multivariate analysis of factors affecting major morbidity after neoadjuvant therapy and esophagectomy.

Variables	*N* (%)	Univariate analysis		Multivariate analysis	
		Unadjusted OR (95% CI)	*p*‐value	Adjusted OR (95% CI)	*p*‐value
Age (years)					
<65	89 (40.3)	1	0.047	1	0.23
≥65	132 (59.7)	1.908 (1.002–3.635)		1.532 (0.765–3.070)	
ASA score					
I/II	207 (93.7)	1	0.037	1	0.15
III/IV	14 (6.3)	3.059 (1.024–9.137)		2.380 (0.739–7.665)	
Diabetes mellitus					
None	194 (87.8)	1	0.022	1	0.026
History	27 (12.2)	2.574 (1.125–5.891)		2.812 (1.132–6.968)	
Operation type					
MIE	175 (79.2)	1	0.026	1	0.10
OE	46 (20.8)	2.170 (1.089–4.323)		1.874 (0.886–3.968)	
eSAS					
>6	105 (47.5)	1	<0.001	1	<0.001
≤6	116 (52.5)	4.485 (2.249–8.947)		3.794 (1.859–7.743)	

*Note*: Data are presented as *n* (%).

Abbreviations: ASA score, American Society of Anesthesiologists score; CI, confidence interval; eSAS, esophagectomy surgical Apgar score; MIE, minimally invasive esophagectomy; OE, open esophagectomy; OR, odds ratio.

**TABLE 4:** Association between eSAS and the detailed complications.

Complication	Number of patients		
	eSAS ≤6 (*N* = 116)	eSAS >6 (*N* = 105)	*p*‐value
Anastomotic leakage			
Absent	99 (85.3)	101 (96.2)	0.006
Present	17 (14.7)	4 (3.8)	
Reintubation			
Absent	104 (89.7)	102 (97.1)	0.027
Present	12 (10.3)	3 (2.9)	
Pneumonia			
Absent	91 (78.4)	90 (85.7)	0.16
Present	25 (21.6)	15 (14.3)	
Surgical site infection			
Absent	105 (90.5)	103 (98.1)	0.017
Present	11 (9.5)	2 (1.9)	
Recurrent nerve paralysis			
Absent	113 (97.4)	99 (94.3)	0.24
Present	3 (2.6)	6 (5.7)	
Sepsis			
Absent	115 (99.1)	105 (100)	0.34
Present	1 (0.9)	0 (0)	
Cardiac complication			
Absent	113 (97.4)	103 (98.1)	0.73
Present	3 (2.6)	2 (1.9)	
Chylothorax			
Absent	114 (98.3)	104 (99)	0.62
Present	2 (1.7)	1 (1)	

*Note*: Data are presented as *n* (%).

Abbreviation: eSAS, esophagectomy surgical Apgar score.

### Survival analysis

**FIGURE 3:**
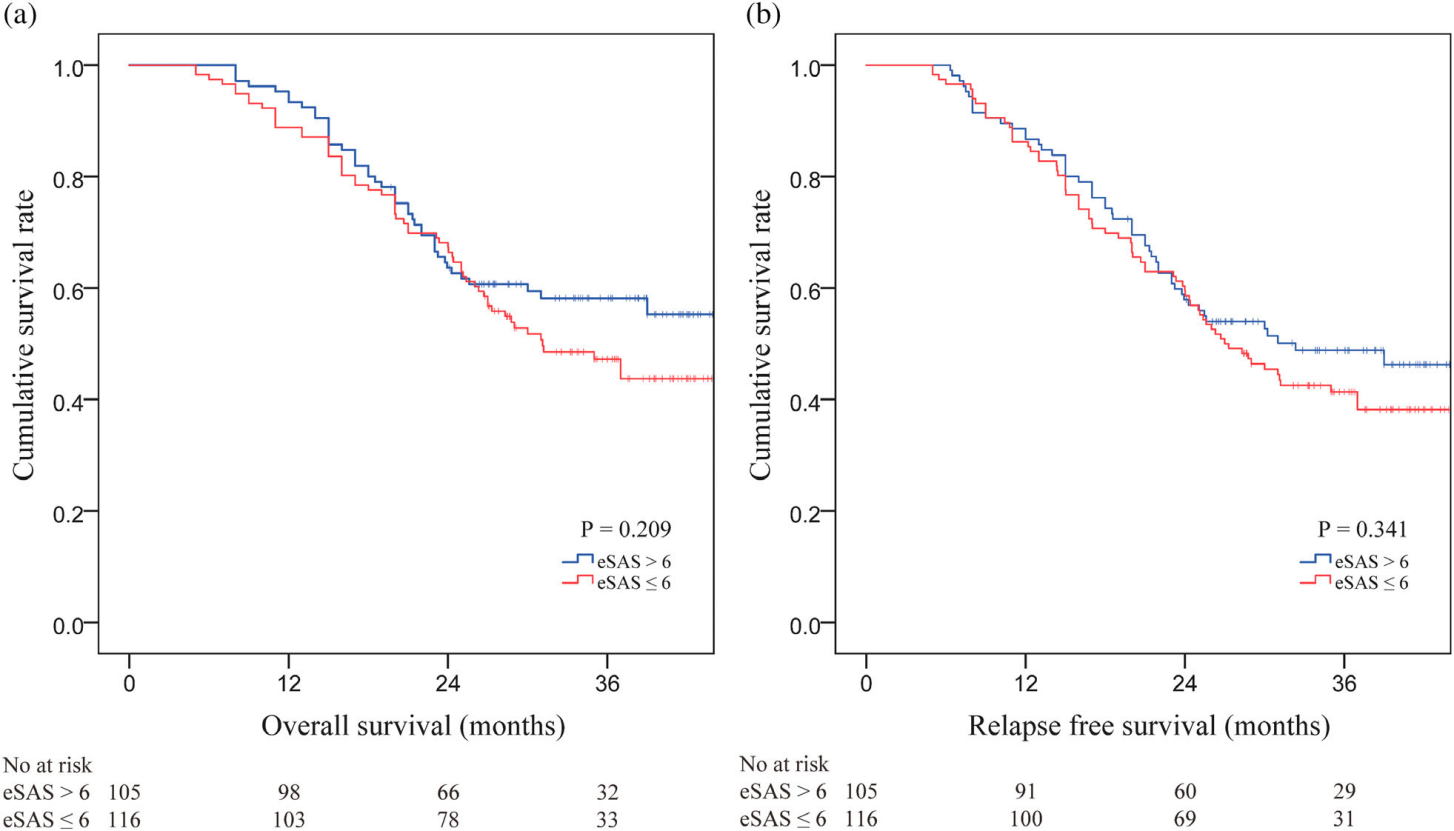
(a) Kaplan–Meier curves of eSAS for overall survival (OS). (b) Kaplan–Meier curves of eSAS for relapse‐free survival (RFS). eSAS, esophagectomy surgical Apgar score.

In total, the average follow‐up duration was 31.9 months (95% CI: 30.2–32.8 months). The patients with eSAS >6 did not have a significant advantage in OS over those with eSAS ≤6 (survival rates during the follow‐up period were 55.1% vs. 43.5%, respectively, *p* = 0.209; Figure 3a). Meanwhile, no prominent advantage in RFS was shown in the eSAS >6 group compared to the eSAS ≤6 group. (46.5 vs. 38.4, *p* = 0.341; Figure 3b).

## DISCUSSION

In this retrospective study, we aimed to evaluate the predictive value of eSAS in EC patients following neoadjuvant therapy and esophagectomy. The results indicated that eSAS score has the potential to serve as a reliable predictor for major morbidity, while its association with prognosis is inconclusive.

Initially introduced by Gawande et al,^26^ the surgical Apgar score (SAS) was devised to appraise surgical procedures in general and vascular operations. However, due to advancements in esophagectomy techniques and the increasing adoption of minimally invasive surgery, intraoperative blood loss has significantly reduced. Thereby, the conventional cutoff value is no longer applicable in the current context. Subsequently, the esophagectomy surgical Apgar score (eSAS) has emerged as a more specific predictor for esophagectomy, particularly for minimally invasive procedures (MIE).^18^ Nevertheless, the predictive capacity of eSAS for perioperative outcomes in esophageal cancer patients remains a subject of debate, with limited studies investigating its relationship with prognosis.

In this retrospective study, we aimed to evaluate the predictive value of eSAS in EC patients following neoadjuvant therapy and esophagectomy. The results indicated that eSAS score has the potential to serve as a reliable predictor for major morbidity. Our study results indicated that eSAS and diabetes mellitus are two independent risk factors for major morbidity, with a lower eSAS significantly correlated with major morbidity, especially anastomotic leakage, surgical site infection and reintubation. Mechanistically, the disease burden of diabetes mellitus leads to hyperglycemia which is an infaust factor for wound healing.^29^, ^30^ However, the underlying biological mechanism linking eSAS to postoperative complications has not been thoroughly investigated. It is widely acknowledged that a low score leads to inadequate blood perfusion and hemodynamic instability. Numerous studies have corroborated the close association between intraoperative hemodynamic parameters included in eSAS and postoperative outcomes.^31^, ^32^, ^33^, ^34^ Therefore, eSAS could serve as a reliable and convenient tool for assessing the hemodynamic status of patients undergoing esophagectomy, facilitating swift prognostic assessments for surgeons. Importantly, eSAS not only enables the prediction of perioperative outcomes but also fosters the quality evaluation. Through the analysis of cases with lower scores, eSAS could identify areas that warrant potential enhancements.^18^ Surgeons might take into consideration modifying surgical techniques or approaches for patients with low scores.

The relationship between eSAS and prognosis was initially studied by Nakagawa et al.,^35^ and he discovered that patients with higher eSAS scroes had significantly lower survival rates than those with lower scores (*p* = 0.043) In contrast, the study by Hayashi et al. did not observe a significant correlation between eSAS and OS (*p* = 0.646) or RFS (*p* = 0.435).^36^ The disparity between their studies might be attributed to variations in the disparity of cutoff points (5 vs. 6) and differences in the follow‐up duration (5 years vs. 3 years). Since stage IA patients were involved in both studies, it is likely that the patients who participated in our study achieved higher clinical stage, as stage IA patients being excluded due to the absence of neoadjuvant therapy. Additionally, poorer prognosis associated with neoadjuvant chemotherapy in the study by Nakagawa et al., as determined by both univariate and multivariate analyses, could explain the lower eSAS scores observed. Meanwhile, our study delineated no significant correlation between low eSAS scores and prognosis (OS, *p* = 0.209; RFS, *p* = 0.341), which is consistent with the findings of Hayashi et al. In their study, low eSAS scores were associated with arterial insufficiency and hypoxia, making them a significant predictive factor for postoperative morbidity, particularly for anastomotic leakage. However, since they did not induce systemic inflammation directly, they could not serve as prognostic factors. Furthermore, we suggest that since eSAS is primarily focused on instantaneous intraoperative hemodynamics, it could provide an insight into predicting perioperative major morbidity. On the other hand, as hemodynamic changes are dynamic in nature, eSAS is unable to reflect the long‐term state accurately. As a result, the OS and RFS could not be predicted.

Certainly, there were several inherent limitations associated with our study. For instance, the sample size was insufficient as this was a retrospective study conducted at a single center, which resulted in the impossibility of subtype analysis of surgical methods and complications. Moreover, due to the limitation of inferior compliance and diversity of the treatment strategy, we could hardly acquire the precise adjuvant therapy of all patients. Taken together, it is crucial to conduct further multicenter clinical studies to explore the correlation and mechanism between eSAS, major morbidity, and OS in patients undergoing neoadjuvant chemotherapy and esophagectomy.

In conclusion, the eSAS score can be used as a predictive factor for major morbidity, especially for anastomotic leakage, surgical site infection and reintubation, in patients with EC who have undergone neoadjuvant therapy and esophagectomy. Furthermore, no significant correlation was observed between the eSAS and OS or RFS. Hence, it is essential to carry out more studies to thoroughly investigate the relationship between eSAS and postoperative morbidity utilizing the same cutoff value in the context of esophagectomy and neoadjuvant therapy.

## AUTHOR CONTRIBUTIONS

Qin Wang and Yi Shen conceived of the idea and were major contributors in writing the manuscript; Zheng Zhang and Qiang Yong collected the data; Qi Chen, Xu Fei and Zhang Chi performed the statistical analysis. All authors contributed to the interpretation of the results and critically reviewed the first draft. All authors read and approved the final manuscript.

## FUNDING INFORMATION

This work was supported by the National Natural Science Foundation of China (81702444) and the Natural Science Foundation of Jiangsu Province (BK20181239).

## CONFLICT OF INTEREST STATEMENT

The authors have no conflicts of interest to declare.

## Data Availability

The data of EC patients in our hospital are not publicly available due to privacy and data sharing issues but are available from the corresponding author on reasonable request.
